# Reproducibility and relative validity of a food frequency questionnaire for a diet-related study in a rural Chinese population

**DOI:** 10.1186/s12937-021-00756-8

**Published:** 2022-02-15

**Authors:** Xudong Liu, Haiyi Li, Yue Zhao, Jun Li, Jianfeng Zhang, Liya Ma, Lin Li, Sihao Lin, Xiang Qian Lao, Wenjing Zhao

**Affiliations:** 1 School of Public Health, Guangdong Pharmaceutical University, No. 283 Jianghai Avenue, Haizhu District, Guangzhou, 510310 China; 2 Department of Epidemiology, School of Public Health, Sun Yat-sen University, Guangzhou, China; 3 Shantou University Medical College, Shantou, China; 4 Department of Cancer Prevention and Treatment, Yanting Cancer Hospital, Mianyang, China; 5 Center of Family Planning Service, Mianyang Maternal and Child Health Care Hospital, Mianyang, China; 6 School of Management, Putian University, Putian, China; 7 JC School of Public Health and Primary Care, The Chinese University of Hong Kong, Hong Kong, SAR China; 8 School of Public Health and Emergency Management, Southern University of Science and Technology, No.1088 Xueyuan Dadao, Nanshan District, Shenzhen, 518055 China

**Keywords:** Food frequency questionnaire, Dietary recall, Validity, Reproducibility, Rural population

## Abstract

**Background:**

This study aimed to assess the reproducibility and validity of a food frequency questionnaire (FFQ) developed for diet-related studies in a rural population.

**Methods:**

One hundred fifty-four healthy residents were interviewed with a 76-item FFQ at baseline (FFQ1) and 1 month later (FFQ2) to assess reproducibility, and required to complete two three-day dietary recalls (DRs) between two FFQs to determine the validity by comparing DRs with FFQ1.

**Results:**

Crude Spearman correlation coefficients between FFQ1 and FFQ2 ranged from 0.58 to 0.92 and energy-adjusted coefficients ranged from 0.62 to 0.92; weighted kappa statistic covered a spectrum from 0.45 to 0.81, depicting moderate to good agreements. For validity, there were moderate to strong associations (0.40–0.68) in most nutrients and food between FFQ1 and DRs; weighted kappa statistic demonstrated fair to moderate agreements for nutrients and food (0.21–0.49).

**Conclusions:**

The results suggest that the FFQ has reasonably reproducibility and validity in measuring most nutrients and food intake, and it can be used to explore the dietary habits in studying the diet-disease relationship in Chinese rural populations.

## Introduction

Food frequency questionnaire (FFQ) is the most widely used method in assessing nutrients and food intake in epidemiological studies. It is a cost-effective and easy-conducted approach in studying a diet-disease association [1–3]. However, food intake varies largely in light of ethnicity, socioeconomic status, diverse lifestyle and cultural background of populations concerned [1]. Each FFQ designed for a specific aim must be effective to obtain true information on individual dietary consumption. A major limitation of using FFQ is measurement errors relating to incomplete food list and the inaccuracies in estimation of intake frequency and portion size [1]. Therefore, examining the reproducibility and validity of FFQ is necessary and crucial in dietary related studies.

In recent years, extensive attention is being paid to the increasing healthy issues of rural population. In China, about half of the population live in rural areas. Though some FFQs had been used to collect dietary information in different Chinese populations [4–14], we did not find a reproducible and validated FFQ suited to population lived in the rural areas of southwest China. Thus, we developed a 76-item FFQ for assessing the habitual diet, and our previous results showed that this FFQ was reasonably reproducible and valid to assess the overall dietary consumption via dietary pattern method in the target rural population [15]. However, the developed FFQ has not yet been appropriately validated for investigating nutrients and food intake, which may make some of the findings of the diet-related studies difficult to interpret [16, 17].

Hence, the objective of this study was to assess the reproducibility and relative validity of the designed FFQ in relation to food and nutrients intake. The reproducibility was tested by comparing the results of two FFQs administered with same interview approach with 1 month apart, and the validity was assessed by comparing intake from the first FFQ and from multiple 24-h dietary recalls.

## Methods

### Study setting and subjects

A total of 196 participants were randomly selected from healthy residents in Yanting County, Southwest China. Inclusion criteria were healthy permanent residents living in local area, male and female, aged from 40 to 70 years. Exclusion criteria were permanent residents with digestive diseases or any type of neoplasm. The sampling frame for all residents aged 40–70 years was available from local government and no significant difference in age and gender was found between recruited and non-recruited participants. This study was conducted according to the guidelines of Declaration of Helsinki and was approved by the Ethical Review Committee for Biomedical Research, School of Public Health, Sun Yat-sen University. Informed written consent was provided by each participant.

### Data collection

**Fig. 1:**
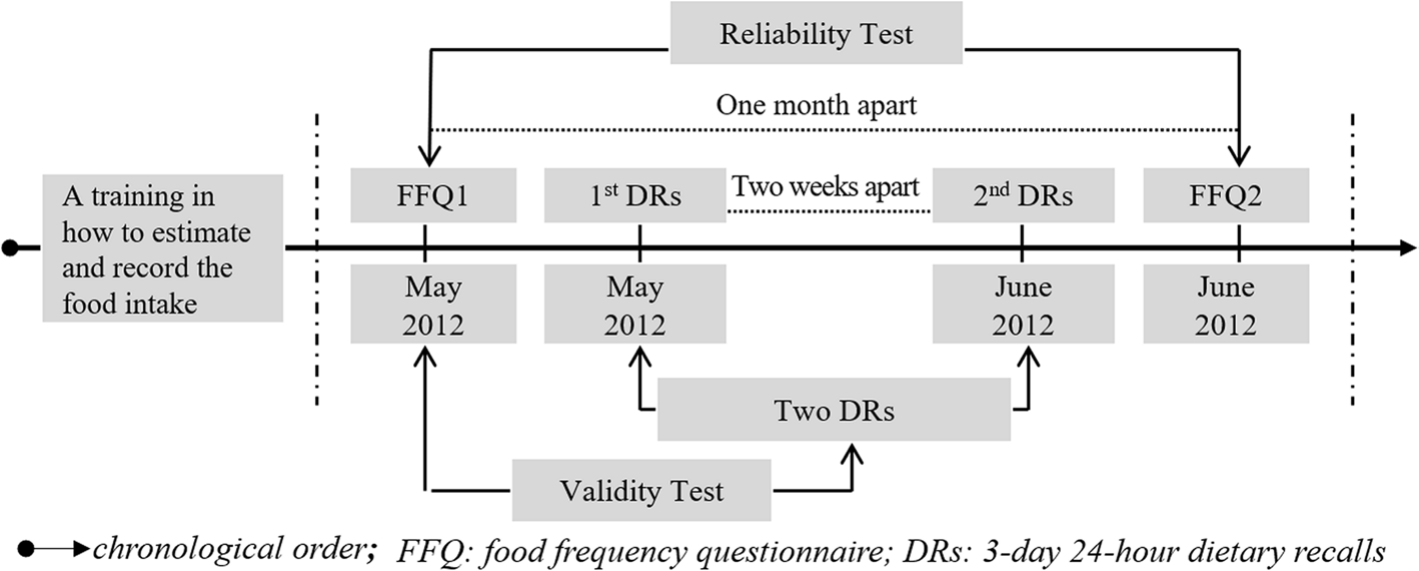
Study design and schedule used in this study. A 76-items food frequency questionnaire (FFQ) was conducted with face-to-face interview at baseline (FFQ1) and 1 month later (FFQ2). Two three-day dietary recalls (DRs) were completed by participants between FFQ1 and FFQ2, with two-week apart. The reproducibility was tested by comparing the results from FFQ1 and FFQ2, and the validity was assessed by comparing results from FFQ1 and from DRs

This study was initiated in May 2012 and ended in June 2012. The study procedure and schedule can be seen in Fig. 1. Before the study, a standardized tool (bowl with four scales inside, i.e., ¼, ½, ¾, and 1), a food photo album and a portable electronic kitchen scale (0.1 g ~ 3 kg, Cameral, China) were provided to each participant. Participants and local recruited interviewers were trained by a registered dietitian in estimating the food weight and recording the frequency and amount in the questionnaires. The ingredients of each mixed dish together with portion sizes required to be recorded in detail.

The first FFQ (FFQ1) and the second FFQ (FFQ2) were conducted at a same rural health clinic, with 1 month apart. Between two FFQs, two 3-day dietary recalls (DRs) were carried out with 2 weeks apart. The information of age, gender, marital status, educational level, weight, height, smoking status, and alcohol drinking was collected by using a structural questionnaire when the first FFQ (FFQ1) was administered. Seven local trained interviewers conducted this study with local dialect. Any unclear records would be corrected by asking the participants to clarify answers and any missing/incomplete information would be checked up.

The FFQ was developed based on a well-known FFQ by National Cancer Institute [18] and based on food availability and local dietary culture in southwest China. A total of 76 food items listed in the FFQ can be seen in our previous report [15]. The FFQ included more than 97.5% of all typical foods that were commonly consumed by the local residents. The FFQ was implemented by trained interviewers with face-to-face interview approach. For each item, participants were asked to recall how frequently they consumed the food or food group in the past 1 year, and a question on the amount consumed each time measured with a standard bowl was followed [19]. The intake amount each time was classified as ≤¼ bowl, ¼-½ bowl, ½-1 bowl, and > 1 bowl for vegetables, meat, soy products and nuts and seeds, as ≤¼ bowl, ¼-½ bowl, ½-1 bowl, 1–2 bowl, 2–3 bowl, and > 3 bowl for cereals and tuber crops, as ≤¼, ¼-½, ½-1,1–2 and > 2 for fruits, fresh eggs and salted eggs. The frequency of food consumption was classified as ≤1 time per month, 1–3 times per month, 1–3 times per week, 4–6 times per week, once per day and more than once per day. For the sake of analysis, 1 month was equal to 4 weeks, and 1 week equal to 7 days. The intake frequency and intake amount each time of each item were re-coded in terms of the mid-point of each category. For example, 1–3 times per month was converted into 2 times per month, and then equated to 0.071 (2 ÷ (4 × 7) = 0.071) times per day; ≤¼ bowl per time was shifted to 0.125 (1/4 ÷ 2 = 0.125) bowl per time; > 1 bowl per time was transferred into 1.5 bowls per time. Then we weighed each bowl of food with a portable electronic kitchen scale, and intake amount each time of each food for each subject was obtained by multiplying the portion size by the weight of each portion. Average daily intake of each item in gram (g) was estimated by multiplying the intake frequency each day by the intake amount each time.

Between the two-FFQ interviews, participants were invited to complete two 3-day dietary recalls questionnaires with 2 weeks apart. In each 3-day dietary recalls, all participants were asked to record all food (including recipes/ingredients of mixed dishes) they consumed from the last day (22:00) to next day (22:00) on the 24-h dietary recall questionnaires in three consecutive days (including two weekdays and one weekend day). A total of 6 days (two weekends and four weekdays) dietary consumption information was collected. Dietary recalls data included single food (such as chicken, egg, and orange) and mixed dishes (such as scrambled egg with tomato). All mixed dishes were converted into the original single food. The weight of each food from mixed dishes was calculated based on the ingredients and their portion sizes recorded on the questionnaires. For example, a participant recorded that he consumed one bowl of scrambled egg with tomato (30% of eggs and 70% of tomato). By using portable electronic kitchen scale, it was found that one bowl of eggs weighed 130 g and one bowl of tomatoes weighed 150 g. Then this participant consumed this dish with 39 g (130 g × 30%) eggs and 105 g (150 g × 70%) tomatoes.

### Statistical analysis

Average daily intake of energy, nutrients and food from two FFQs and dietary recalls was analysed, respectively, by using CDGSS 3.0 software [20] updated with latest Food Components Databases [21, 22]. Then the food items were grouped according to their natural similarities. Mean with standard deviation (S.D.) was used to describe the distributions of average daily intake of energy, nutrients, and food. Energy-adjusted intakes of food and nutrients were calculated by using residual method [23] to remove the variation caused by energy intake and were used to calculate correlation coefficients. The reproducibility was assessed by means of comparing average daily intakes of nutrients and food from FFQ1 with those from FFQ2. The validity was assessed by comparing average daily intake of nutrients and food from FFQ1 with those from dietary recalls. As the original and log_10_-transformed data did not comply with a normal distribution according to one sample K-S test, paired Wilcoxon signed rank test was used to compare the difference of average daily intake of nutrients and food, and Spearman rank correlation coefficients were used to access the association between average daily intakes of nutrients and food [24]. The correlation coefficients of 0.10–0.39, 0.40–0.69, 0.70–0.89, and 0.90–1.00 represents week, moderate, strong and very strong correlation, respectively [25]. The average daily intake of food and nutrients were divided into tertile, then we can assess inter-rate agreement by using weighted kappa (κ) statistic [26] and assess the percentages of misclassification and agreement according to the Masson and colleagues criteria [27]. A weighted kappa statistic value of ≤0.2, 0.21–0.40, 0.41–0.60, 0.61–0.80, and ≥ 0.81 represents slight, fair, moderate, substantial, and good agreement, respectively [28]. Statistical analyses were performed using SPSS (version 19.0, 2010, IBM SPSS Inc). A two-sided *p*-value ≤0.05 was considered statistically significant.

## Results

One hundred eighty out of 196 residents (91.8%) agreed to take part in this one-month follow-up study. During the follow-up, nine participants moved to other regions, fifteen participants who did not provide completed dietary recall questionnaire, and two participants who reported implausible energy intake (> 8000 kcal/day) were excluded from the data analysis. Therefore, 154 participants who completed two FFQs and multiple dietary recalls were included in the following analysis.

Table 1 shows the characteristics of the 154 rural participants included in this study. The mean (S.D.) age of the participants was 54.1 (8.4) years, ranging from 40 to 69 years old. The mean (S.D.) of body mass index was 23.9 (3.4) kg/m^2^. More than half (59%) of the participants were males and the majority (95.5%) were married. 64.3 and 58.4% of participants had habits of tobacco smoking and alcohol drinking, respectively (Table 1). The mean values of most nutrients and food intake derived from FFQ1 were approximately equal to those from FFQ2, except for retinol, calcium, and tuber crops (Table 2); the mean values of most food and nutrients derived from dietary records were approximately equal to those from FFQ1, except for tuber crops, fruits, white meat, carotene, and retinol.

**Table 1:** Characteristics of participants in the study

Basic characteristics	Value
Age, years, mean (SD)	54.1 (8.4)
Body mass index, kg/m^2^, mean (SD)	23.9 (3.4)
Gender, N (%)	
Male	91 (59.1)
Female	63 (40.9)
Education, N (%)	
Primary school and below	75 (48.7)
Middle school and above	79 (51.3)
Married, N (%)	
Married	147 (95.5)
Divorced and others	7 (4.5)
Smoking status, N (%)	
No	99 (64.3)
Yes	54 (35.7)
Alcohol drinking, N (%)	
No	90 (58.4)
Yes	64 (41.6)

**Table 2:** Average daily intake of nutrients and food intake from FFQ1, FFQ2 and dietary recalls

Items ^a,b^	FFQ1 ^a,b^	FFQ2 ^a,b^	DRs ^a,b^
Energy (MJ)	10.6 (3.2)	9.7 (2.7)	9.6 (4.6)
Protein (g)	71.7 (22.5)	65.1 (18.5)	65.2 (64.8)
Fat (g)	38.4 (18.5)	33.3 (13.3)	38.4 (38.9)
Fiber (g)	12.6 (4.9)	11.3 (4.2)	11.0 (423.0)
Carbohydrate(g)	473.0 (154.6)	438.3 (128.5)	423.9 (10.7)
Carotene (μg)	1098.9 (675.3) ^c^	1077.7 (579.3)	1256.2 (1251.8)
Retinol equivalent (μg)	299.5 (147.7) ^c, d^	249.9 (122.3)	329.5 (330.4)
Thiamine (mg)	1.6 (0.5)	1.4 (0.5)	1.4 (1.4)
Riboflavin (mg)	0.7 (0.2)	0.6 (0.2)	0.7 (0.7)
Niacin (mg)	21.8 (7.1)	19.6 (5.6)	19.3 (19.1)
Vitamin C (mg)	45.9 (31.0)	37.0 (26.5)	54.1 (53.6)
Vitamin E (mg)	10.6 (5.4)	9.2 (4.1)	8.2 (7.9)
Calcium (mg)	238.2 (98.0) ^d^	204.1 (74.5)	203.7 (201.9)
Iron (mg)	27.6 (8.2)	24.8 (6.4)	23.0 (23.0)
Zinc (mg)	10.8 (3.6)	9.9 (3.1)	10.2 (10.1)
Selenium (mg)	19.5 (8.3)	17.2 (6.3)	20.2 (20.0)
Eggs (g)	22.7 (23.8)	21.2 (31.0)	16.9 (12.8)
Red meat(g)	43.5 (24.9)	39.4 (32.9)	59.3 (15.4)
White meat (g)	2.2 (4.0) ^c^	2.3 (3.3)	4.7 (8.2)
Soy products (g)	30.3 (30.6)	32.4 (32.2)	23.1 (16.0)
Fresh vegetables (g)	106.2 (93.2)	95.2 (77.0)	100.9 (23.2)
Processed vegetables (g)	14.1 (18.9)	12.4 (15.2)	20.3 (21.5)
Fruits (g)	49.6 (49.2) ^c^	52.3 (54.7)	21.7 (15.7)
Nuts and seeds (g)	15.0 (15.7)	14.4 (21.8)	11.1 (5.1)
Cereals (g)	359.1 (107.9)	355.0 (86.9)	337.5 (71.5)
Tuber crops (g)	28.1 (40.1) ^c, d^	21.7 (29.5)	16.9 (23.9)

^a^Average daily intake of nutrients and food: mean and standard deviation (S.D.)

^b^*Abbreviation: FFQ1,* the first survey of food frequency questionnaire; *FFQ2,* the second survey of food frequency questionnaire; *DRs,* two three-day dietary recalls;

^c^Different from DRs, comparing average daily intake, Paired Wilcoxon Rank Test, *P* < 0.05

^d^Different from FFQ2, comparing average daily intake, Paired Wilcoxon Rank Test, *P* < 0.05

The comparison of FFQ1 with FFQ2 is shown in Table 3. Between the two FFQs, the crude Spearman rank correlation coefficients ranged from 0.58 (nuts and seeds) to 0.92 (fruits) and energy-adjusted correlation coefficients ranged from 0.62 (nuts and seeds) to 0.92 (fruits). The proportion of participants classified into same tertile of both FFQ1 and FFQ2 ranged from 55.45% (tuber crops) to 86.36% (processed vegetables) and the percentage of participants into extreme tertile ranged from 0.65% (fresh vegetables and processed vegetables) to 7.79% (carotene and iron). The weighted *k* statistic between the two FFQs ranged from 0.45 (soy products) to 0.81(fruits).

**Table 3:** Spearman correlation coefficients, percentage of agreement and weighted kappa statistic between FFQ1 and FFQ2

	Spearman correlation coefficients		Percentage of agreement (%)			κ^a *c*^
	Crude ^b^	Energy adjusted ^b^	Same tertile	Adjacent tertile	Extreme tertile	
Energy	0.73		69.48	27.27	3.25	0.62
Protein	0.75	0.73	64.94	32.47	2.60	0.58
Fat	0.75	0.70	66.23	31.17	2.60	0.59
Fiber	0.74	0.73	59.09	37.01	3.90	0.50
Carbohydrate	0.74	0.71	71.43	24.68	3.90	0.63
Carotene	0.72	0.71	59.74	32.47	7.79	0.46
Retinol	0.77	0.74	64.94	32.47	2.60	0.58
Thiamine	0.79	0.71	67.53	30.52	1.95	0.61
Riboflavin	0.79	0.79	68.18	30.52	1.30	0.63
Niacin	0.66	0.63	64.94	31.17	3.90	0.56
Vitamin C	0.70	0.70	58.44	35.06	6.49	0.46
Vitamin E	0.71	0.74	68.83	27.92	3.25	0.61
Calcium	0.68	0.71	57.79	38.96	3.25	0.49
Iron	0.61	0.71	64.94	27.28	7.79	0.52
Zinc	0.79	0.65	66.88	29.87	3.25	0.59
Selenium	0.81	0.76	69.48	27.27	3.25	0.62
Eggs	0.87	0.86	79.87	18.18	1.95	0.75
Red meat	0.82	0.71	80.52	16.23	3.25	0.74
White meat	0.79	0.65	67.78	29.22	3.00	0.53
Soy products	0.80	0.80	79.37	15.87	4.76	0.45
Fresh vegetables	0.69	0.68	63.64	31.82	4.55	0.56
Processed vegetables	0.82	0.83	86.36	12.99	0.65	0.72
Fruits	0.92	0.92	83.77	15.58	0.65	0.81
Nuts and seeds	0.58	0.62	59.74	38.31	1.95	0.47
Cereals	0.74	0.68	68.83	27.27	3.90	0.60
Tuber crop	0.70	0.64	55.45	38.86	5.69	0.46

^a^*Abbreviation*: *FFQ1,* the first survey of food frequency questionnaire; *FFQ2,* the second survey of food frequency questionnaire; *κ* statistics, for weighted kappa test;

^b^For Crude and energy-adjusted Spearman correlation coefficients, all values were significant (*P* < 0.05)

^c^All weighted kappa coefficients were significant (*P* < 0.05) except for vitamin E and white meat

The comparison of FFQ1 with dietary records is shown in Table 4. The crude Spearman correlation coefficients between FFQ1 and dietary records ranged from 0.25 (white meat) to 0.66 (fruits), and energy-adjusted correlation coefficients ranged from 0.21 (white meat) to 0.68 (iron). The percentage of participants classified into the same tertile ranged from 45.45% (nuts and seeds) to 59.09% (vitamin E), while the percentage of participants into extreme tertile ranged from 3.25% (cereals) to 14.29% (nuts and seeds). Weighted *k* statistic ranged from 0.21 (white meat) to 0.49 (vitamin E).

**Table 4:** Spearman correlation coefficients, percentage of agreement and weighted kappa (κ) statistic of daily intake of nutrients and food between FFQ1 and DRs

	Spearman correlation coefficients		Percentage of agreement (%)			κ^a d^
	crude ^b^	Energy adjusted ^c^	Same tertile	Adjacent tertile	Extreme tertile	
Energy	0.55	–	50.75	45.35	3.90	0.36
Protein	0.44	0.43	50.75	40.16	9.09	0.30
Fat	0.45	0.41	50.40	41.16	8.44	0.31
Fiber	0.58	0.54	53.90	39.61	6.49	0.41
Carbohydrate	0.45	0.45	50.40	41.16	8.44	0.31
Carotene	0.54	0.54	51.30	40.26	8.44	0.36
Retinol	0.45	0.45	50.51	41.05	8.44	0.30
Thiamine	0.40	0.53	53.21	39.05	7.74	0.34
Riboflavin	0.39	0.39	48.10	42.81	9.09	0.29
Niacin	0.45	0.41	50.65	42.86	6.49	0.37
Vitamin C	0.51	0.50	53.90	37.66	8.44	0.38
Vitamin E	0.65	0.50	59.09	36.36	4.55	0.49
Calcium	0.57	0.50	53.90	38.96	7.14	0.40
Iron	0.43	0.68	50.75	39.86	9.39	0.28
Zinc	0.40	0.47	47.40	44.16	8.44	0.31
Selenium	0.43	0.39	48.05	42.86	9.09	0.31
Eggs	0.46	0.44	52.40	41.75	5.84	0.34
Red meat	0.40	0.42	51.21	39.75	9.04	0.32
White meat	0.25	0.21	49.35	39.61	11.04	0.21
Soy products	0.61	0.48	58.73	31.75	9.52	0.36
Fresh vegetables	0.49	0.46	53.90	37.66	8.44	0.38
Processed vegetables	0.42	0.40	51.01	39.45	9.53	0.31
Fruits	0.66	0.62	53.25	40.26	6.49	0.41
Nuts and seeds	0.44	0.41	45.45	40.26	14.29	0.22
Cereals	0.56	0.61	55.84	40.91	3.25	0.47
Tuber crop	0.46	0.49	50.51	39.66	9.83	0.32

^a^*Abbreviation*: *FFQ1,* the first pass of food frequency questionnaire, *DRs,* two 3-day dietary recalls; *κ,* statistic for weighted kappa test;

^b^All crude coefficients were significant (*P* < 0.05) except for white meat

^c^All adjusted coefficients were significant (*P* < 0.05) except for white meat

^d^All weighted Kappa values were significant (*P* < 0.05) except for white meat and riboflavin

## Discussion

This report showed the reproducibility and validity of an FFQ designed to capture the common intake of nutrients and major food in a rural Chinese population. The results demonstrated that the FFQ had reasonable reproducibility (correlation coefficients ≥0.58 and weighted κ statistic > 0.45) for all selected food and nutrients and fair to moderate validity (correlation coefficients > 0.40 and weighted κ coefficients > 0.3) for most of the food and nutrients.

The means of some nutrients and food from FFQ1 were slightly higher than those from FFQ2. However, no significant difference was found for most items (except for retinol), indicating the learning effect was not a major concern. In China, people tended to mix several food items together, which made it difficult to estimate the accurate amount of each item, and they might overestimate the intake of some items when FFQ was used. However, a noteworthy difference between FFQ1 and dietary records was only seen in tuber crops, fruits, white meat, carotene, and retinol, indicating the overestimation in FFQ did not happen in most items.

In this study, the dietary intake survey with FFQ was conducted twice with 1 month apart to test the reproducibility of FFQ, which was similar to other reports [29–31]. There would be an overlap between FFQ1 and FFQ2 as they were finished a month apart reflecting an 11-month overlap in recall time; however, two FFQ surveys were done to examine the reproducibility, and like many other studies [1, 29, 31], the overlap could not significantly affect the results. This interval could be long enough for participants to forget their previous responses, but short enough for participants not to change their dietary and life habits [2]. The length of FFQ and the number of food items should be decided based on objective of the study, food accessibility and variability of food consumption in the target population [1, 2]. In this study, the eating habits and lifestyle of residents were not changed over time as much as many other Chinese people did. We selected most consumed dietary items, covering more than 97.5% of typical food in the region, which could reflect the usual dietary habits.

In testing the reproducibility, both crude and adjusted Spearman correlation coefficients showed that FFQ1 and FFQ2 were moderately to strongly correlated in macronutrients (0.70–0.75), micronutrients (0.61–0.81) and food (0.58–0.92). The correlation coefficients in this study were higher than those in other Chinese studies [4–9, 13, 14], this might due to the fact that most of Chinese studies adopted an interval of 9 to 24 months when testing the reproducibility of FFQs, which might increase the risk of changing dietary habits. Masson and colleagues’ criteria require that more than 50% of participants should be correctly classified into same tertile and less than 10% into the opposite tertile [27]. In this study, the results showed that more than 50% of participants were correctly classified into same tertile and less than 8% into an opposite tertile, which indicated a reasonably good agreement and less misclassification for all food and nutrients. Weighted *k* statistic further displayed moderate to good inter-rate agreements (0.45–0.81) for all food and nutrients [26]. The dietary consumption in the population concerned lacked diversity. More often, the type and quantity of food consumed by local residents kept consistent and did not change in a relative long period [32], this might also be the explanation for stronger correlations and better agreement in food and nutrients between two FFQs.

Many factors may influence the evaluation of validity, such as reference method, days of diet tracked, record period, and the homogeneity of intake within participants [33]. Dietary recall usually represents an optimal comparison method in measuring food intake, because sources of errors from dietary recalls are largely independent errors associated with a food frequency questionnaire [1, 2]. Some researchers suggested the optimal study design of dietary record rarely required more than four- or five-day dietary recalls for each participant [2, 34]. In this study, we collected two three-consecutive-day dietary recalls, which have some advantages to explore the day-to-day intake variation. However, this short interval cannot avoid the seasonal/monthly variations in food consumption. This may be the major reason why the correlation coefficients and kappa statistics in some nutrients and food were relatively low between dietary records and FFQ1.

The validity assessment of FFQ in this study was assessed by comparing food and nutrients intake from FFQ1 with those from dietary records. This could avoid some extra influence (such as learning effects [6]) and it was easier to explain the results. Between FFQ1 and dietary records, there were moderate correlations for energy (0.55) and macronutrients (0.41–0.58) and moderate correlations for most micronutrients and food (0.40–0.68), though the correlation coefficients for a few of micronutrients (riboflavin and selenium) and food (white meat, nuts, and seeds) were less than 0.40. Compared with other studies that used the same approach with ours, the correlation coefficients in this study were similar to or larger than those in other areas of China [6–8, 11, 13, 14]. The Spearman correlation coefficients in food items and nutrients decreased when adjusting for energy, which might be due to high inter-person variation in the frequency and amount of food intakes in the study subjects. For most nutrients and food, the percentage of participants correctly classified into same tertile was higher than 50%, which indicated a higher agreement between FFQ1 and dietary records according to the Masson and colleagues’ criteria [27]. In addition, the percentages of participants classified into opposite tertile were lower than 10% for most nutrients and food, apart from white meat, and nuts and seeds, which indicated that the misclassification between FFQ1 and dietary records was small. Compared with results from other studies, the percentages of agreement were similar to studies in Taiwan and some western countries [35–38] and higher than in Belgian (32–76%) [39] and Australia (35–54%) [40]. Meanwhile, the misclassification in most items was lower than those in Taiwan and some western countries [36–41]. Weighted *k* statistic demonstrated a consistent moderate agreement in fiber, vitamin E, calcium, cereals, and fruits (0.40–0.49), fair agreement for most food and nutrients (0.30–0.38), as well as fair agreement in riboflavin, iron, white meat and nuts and seeds (0.21–0.29). Weighted *k* statistic (0.21–0.49) in this study was similar to those in Britain (0.23–0.66) [27] and Belgian (0.10–0.71, 39], which indicated acceptable inter-rater agreements.

We found that there was a weak association and/or low agreement between FFQ1 and dietary records for a few of food and nutrients, especially for white meat, nuts and seeds. The mean of white meat intake from FFQ1 (2.2 g/day) was much lower than that from dietary records (4.7 g/d). This might be due to that the dietary recall method was self-administrated with open-questions, whereas the FFQ was interviewed with in-person approach and with close-ended questions. Although the errors from FFQs and dietary recalls were independent and dietary recall was suggested to be an adequate comparison method for the target instrument [42], self-monitoring of food intake in dietary recalls may lead to eating behavior changes and may make participants pay more attention to their dietary behaviors. The participants might consume more white meat or overestimate white meat intake during the period of recording dietary diary. However, the mean of white meat intake from the FFQ1 in this study was approximate to those reported in another study [32] in a similar population, which tracked food intake in 1 year and found lower intake of white meat (3 g/d ay). This suggested that the FFQ could reasonably reflect yearly white meat intake. There was a lower agreement in the consumption of nuts and seeds between FFQ1 and dietary records. Cross classification analysis classified the participants close to cutoff points into different tertiles. It may increase the percentage of participants classified into the opposite tertile and lower the weighted *k* statistic. Another reason may be that 6 days dietary recalls may not reflect yearly consumption of nuts and seeds, because nuts and seeds consumption has seasonal variation in rural areas [32]. However, there was no significant difference in nuts and seeds intake between FFQ1 and dietary records. Moreover, the mean of nuts and seeds intake from FFQ1 in this study was approximate to that in Chinese adults [43] and in the same targe population [32], which showed that the FFQ in some degree can reflect the consumption of nuts and seeds.

The major strengths of this study include multiple tools or approaches adopted in the estimation of portion sizes in data collection, higher participation rate and the ability to recruit a relative representative sample. However, we acknowledged that two three-day dietary recalls might not be adequate to reflect the seasonal effects and other poorly defined fluctuations in dietary consumption. This is first limitation in this study. Nonetheless, dietary records covered 4 weekdays and 2 weekends, which to some extent could capture the day-to-day variation. The second limitation is that sample size in this study was relatively small which may lower the statistic power. The last limitation is that this study only assessed the relative validity of FFQ by using the dietary recalls, but instead of criterion validity by using biomarkers of dietary exposure.

## Conclusions

The results of this present study suggest that the FFQ has reasonably reproducibility and fair to moderate validity in measuring most nutrients and food intake among the concerned population, and it can be used to explore the dietary habits in studying the diet-disease relationship in Chinese rural populations.

## Data Availability

The data used to support the findings of this study are available from the corresponding author upon request.

## Abbreviations

FFQ: Food frequency questionnaire
DRs: Dietary recalls
κ: Kappa
S.D.: Standard deviation

## References

[1] Willett W (2013). Nutritional epidemiology. 3rd edn. New York.

[2] Cade J, Thompson R, Burley V, Warm D (2002). Development, validation and utilisation of food-frequency questionnaires - a review. Public Health Nutr.

[3] Lee RD, Nieman DC (2007). Nutritional assessment.

[4] Zhang CX, Ho SC (2009). Validity and reproducibility of a food frequency questionnaire among Chinese women in Guangdong province. Asia Pac J Clin Nutr.

[5] Shu XO, Yang G, Jin F, Liu D, Kushi L, Wen W, Gao YT, Zheng W (2004). Validity and reproducibility of the food frequency questionnaire used in the Shanghai Women's health study. Eur J Clin Nutr.

[6] Xia W, Sun C, Zhang L, Zhang X, Wang J, Wang H, Wu L (2011). Reproducibility and relative validity of a food frequency questionnaire developed for female adolescents in Suihua. North China PLoS One.

[7] Zhuang M, Yuan Z, Lin L, Hu B, Wang X, Yang Y, Chen X, Jin L, Lu M, Ye W (2012). Reproducibility and relative validity of a food frequency questionnaire developed for adults in Taizhou. China PLoS One.

[8] Wang X, Sa R, Yan H (2008). Validity and reproducibility of a food frequency questionnaire designed for residents in North China. Asia Pac J Clin Nutr.

[9] Wang X, Yan H, Sa R (2009). Study of validity and reproducibility of food frequency questionnaires for residents over 50-years-old in Xi’an City. Wei Sheng Yan Jiu.

[10] Jian L, Binns CW, Lee AH (2006). Validity of a food-frequency questionnaire for elderly men in Southeast China. Public Health Nutr.

[11] Ke L, Toshiro T, Fengyan S, Ping Y, Xiaoling D, Kazuo T (2005). Relative validity of a semi-quantitative food frequency questionnaire versus 3 day weighed diet records in middle-aged inhabitants in Chaoshan area, China. Asian Pac J Cancer Prev.

[12] Villegas R, Yang G, Liu D, Xiang YB, Cai H, Zheng W, Shu XO (2007). Validity and reproducibility of the food-frequency questionnaire used in the Shanghai men's health study. Br J Nutr.

[13] Li M, Halldorsson TI, Bjerregaard AA, Che Y, Mao YY, Hu WF, Wang Y, Zhou WJ, Olsen SF, Strom M (2014). Relative validity and reproducibility of a food frequency questionnaire used in pregnant women from a rural area of China. Acta Obstet Gynecol Scand.

[14] Cheng Y, Yan H, Dibley MJ, Shen Y, Li Q, Zeng LX (2008). Validity and reproducibility of a semi-quantitative food frequency questionnaire for use among pregnant women in rural China. Asia Pac J Clin Nutr.

[15] Liu X, Wang X, Lin S, Song Q, Lao X, Yu IT (2015). Reproducibility and validity of a food frequency questionnaire for assessing dietary consumption via the dietary pattern method in a Chinese rural population. PLoS One.

[16] Lin S, Wang X, Huang C, Liu X, Zhao J, Yu IT, et al. Consumption of salted meat and its interactions with alcohol drinking and tobacco smoking on esophageal squamous-cell carcinoma. Int J Cancer. 2014. 10.1002/ijc.29406. 10.1002/ijc.29406 25544988

[17] Song Q, Wang X, Yu IT, Huang C, Zhou X, Li J, Wang D (2012). Processed food consumption and risk of esophageal squamous cell carcinoma: a case-control study in a high risk area. Cancer Sci.

[18] Diet History Questionnaire (Version 2.0) [Available: http://appliedresearch.cancer.gov/archive/dhq2/dhq1.2007.sample.pdf].

[19] Kowalkowska J, Wadolowska L, Hamulka J, Wojtas N, Czlapka-Matyasik M, Kozirok W, Bronkowska M, Sadowska J, Naliwajko S, Dziaduch I (2019). Reproducibility of a short-form, multicomponent dietary questionnaire to assess food frequency consumption, nutrition knowledge, and lifestyle (SF-FFQ4PolishChildren) in polish children and adolescents. Nutrients.

[20] Ma L, Grann K, Li M, Jiang Z (2011). A pilot study to evaluate the effect of soy isolate protein on the serum lipid profile and other potential cardiovascular risk markers in moderately hypercholesterolemic Chinese adults. Ecol Food Nutr.

[21] Yang YX, Wang GY, Pan XC (2009). China food composition (book 1.2nd edition).

[22] Yang YX (2004). China food composition (book 2).

[23] Willett WC, Howe GR, Kushi LH (1997). Adjustment for total energy intake in epidemiologic studies. Am J Clin Nutr.

[24] Shrout PE (1998). Measurement reliability and agreement in psychiatry. Stat Methods Med Res.

[25] Schober P, Boer C, Schwarte LA (2018). Correlation coefficients: appropriate use and interpretation. Anesth Analg.

[26] Altman DG (1991). Practical statistics for medical research.

[27] Masson LF, McNeill G, Tomany JO, Simpson JA, Peace HS, Wei L, Grubb DA, Bolton-Smith C (2003). Statistical approaches for assessing the relative validity of a food-frequency questionnaire: use of correlation coefficients and the kappa statistic. Public Health Nutr.

[28] Viera AJ, Garrett JM (2005). Understanding interobserver agreement: the kappa statistic. Fam Med.

[29] Boucher B, Cotterchio M, Kreiger N, Nadalin V, Block T, Block G (2006). Validity and reliability of the Block98 food-frequency questionnaire in a sample of Canadian women. Public Health Nutr.

[30] Barrat E, Aubineau N, Maillot M, Derbord E, Barthes P, Lescuyer JF, et al. Repeatability and relative validity of a quantitative food-frequency questionnaire among French adults. Food Nutr Res. 2012;56. 10.3402/fnr.v56i0.18472 PMC3485403 23118710

[31] Kobayashi T, Kamimura M, Imai S, Toji C, Okamoto N, Fukui M, Date C (2011). Reproducibility and validity of the food frequency questionnaire for estimating habitual dietary intake in children and adolescents. Nutr J.

[32] Xiao P, Tao D, Huang C, Zheng S, Wang H, Du H (2006). Analysis on dietary structure for residents in high-incidence area of esophageal cancer (in Chinese). Modern Prev Med.

[33] Block G, Woods M, Potosky A, Clifford C (1990). Validation of a self-administered diet history questionnaire using multiple diet records. J Clin Epidemiol.

[34] Stram DO, Longnecker MP, Shames L, Kolonel LN, Wilkens LR, Pike MC, Henderson BE (1995). Cost-efficient design of a diet validation study. Am J Epidemiol.

[35] Grootenhuis PA, Westenbrink S, Sie CM, de Neeling JN, Kok FJ, Bouter LM (1995). A semiquantitative food frequency questionnaire for use in epidemiologic research among the elderly: validation by comparison with dietary history. J Clin Epidemiol.

[36] Lee MS, Pan WH, Liu KL, Yu MS (2006). Reproducibility and validity of a Chinese food frequency questionnaire used in Taiwan. Asia Pac J Clin Nutr.

[37] Petkeviciene J, Simila M, Becker W, Kriaucioniene V, Valsta LM (2009). Validity and reproducibility of the NORBAGREEN food frequency questionnaire. Eur J Clin Nutr.

[38] Marques-Vidal P, Ross A, Wynn E, Rezzi S, Paccaud F, Decarli B. Reproducibility and relative validity of a food-frequency questionnaire for French-speaking Swiss adults. Food Nutr Res. 2011;55. 10.3402/fnr.v55i0.5905 PMC3091846 21562629

[39] De Keyzer W, Dekkers A, Van Vlaslaer V, Ottevaere C, Van Oyen H, De Henauw S, Huybrechts I (2013). Relative validity of a short qualitative food frequency questionnaire for use in food consumption surveys. Eur J Pub Health.

[40] Ambrosini GL, de Klerk NH, O'Sullivan TA, Beilin LJ, Oddy WH (2009). The reliability of a food frequency questionnaire for use among adolescents. Eur J Clin Nutr.

[41] Mullie P, Clarys P, Hulens M, Vansant G (2009). Reproducibility and validity of a semiquantitative food frequency questionnaire among military men. Mil Med.

[42] Haftenberger M, Heuer T, Heidemann C, Kube F, Krems C, Mensink GB (2010). Relative validation of a food frequency questionnaire for national health and nutrition monitoring. Nutr J.

[43] Fan TY, Liu AL, He YN, Yang XG, Xu GF, Ma GS (2012). Assessment of nutrients adequacy of adult residents in China. Acta Nutrimenta Sinica.

